# Supplementary material for: DNA sequence and chromatin modifiers cooperate to confer epigenetic bistability at imprinting control regions
Source: Nat Genet. 2022 Nov 4;54(11):1702–10. doi: 10.1038/s41588-022-01210-z (PMC9649441; doi:10.1038/s41588-022-01210-z)
Supplement: Supplementary file 1 [file 41588_2022_1210_MOESM1_ESM.pdf]

nature portfolio

Corresponding author(s): Tuncay Baubec

Last updated by author(s): Jul 22, 2022

Reporting Summary

Nature Portfolio wishes to improve the reproducibility of the work that we publish. This form provides structure for consistency and transparency in reporting. For further information on Nature Portfolio policies, see our Editorial Policies and the Editorial Policy Checklist.

Statistics

For all statistical analyses, confirm that the following items are present in the figure legend, table legend, main text, or Methods section.

n/a	Confirmed	
☐	☒	The exact sample size (*n*) for each experimental group/condition, given as a discrete number and unit of measurement
☐	☒	A statement on whether measurements were taken from distinct samples or whether the same sample was measured repeatedly
☐	☒	The statistical test(s) used AND whether they are one- or two-sided<br>*Only common tests should be described solely by name; describe more complex techniques in the Methods section.*
☒	☐	A description of all covariates tested
☐	☒	A description of any assumptions or corrections, such as tests of normality and adjustment for multiple comparisons
☐	☒	A full description of the statistical parameters including central tendency (e.g. means) or other basic estimates (e.g. regression coefficient) AND variation (e.g. standard deviation) or associated estimates of uncertainty (e.g. confidence intervals)
☐	☒	For null hypothesis testing, the test statistic (e.g. *F*, *t*, *r*) with confidence intervals, effect sizes, degrees of freedom and *P* value noted<br>*Give P values as exact values whenever suitable.*
☒	☐	For Bayesian analysis, information on the choice of priors and Markov chain Monte Carlo settings
☒	☐	For hierarchical and complex designs, identification of the appropriate level for tests and full reporting of outcomes
☒	☐	Estimates of effect sizes (e.g. Cohen's *d*, Pearson's *r*), indicating how they were calculated

Our web collection on statistics for biologists contains articles on many of the points above.

Software and code

Policy information about availability of computer code

Data collection	For data collection we used standard operating software installed on Illumina MiSeq/NovaSeq sequencers, BD Biosciences FACSCanto II cytometer, BD Bioscience FACSAria III cell sorter, and Thermo Scientific Orbitrap Fusion masspectrometers.
Data analysis	For data analysis, the following publicly available tools have been used:<br>FlowJo (10.7)<br>BD FACSDiva (9.1.2)<br>R (4.0.3)<br>BPNet (0.0.22)<br>bowtie 2 (2.3.5.1)<br>trim_galore (0.6.6)<br>picard (2.23.9)<br>MACS2 (2.1.1.20160309)<br>Bismark (0.23.0)<br>Prism (5.0a)<br>QuasR (1.30.0)<br>bedtools (2.27.1)<br>MAGeCK (0.5.9.2)<br>MaxQuant (1.5.3.30)

For manuscripts utilizing custom algorithms or software that are central to the research but not yet described in published literature, software must be made available to editors and reviewers. We strongly encourage code deposition in a community repository (e.g. GitHub). See the Nature Portfolio guidelines for submitting code & software for further information.

Data

Policy information about availability of data

All manuscripts must include a data availability statement. This statement should provide the following information, where applicable:

- Accession codes, unique identifiers, or web links for publicly available datasets
- A description of any restrictions on data availability
- For clinical datasets or third party data, please ensure that the statement adheres to our policy

Following published datasets were used in this study:
WGBS - https://www.ncbi.nlm.nih.gov/geo/query/acc.cgi?acc=GSE30206
H3K9me3, H3K4me3, H3K36me3 and H3K27me3 - https://www.ncbi.nlm.nih.gov/geo/query/acc.cgi?acc=GSE23943
DNase-seq and RNA-seq - https://www.ncbi.nlm.nih.gov/geo/query/acc.cgi?acc=GSE67867
SETDB1 - https://www.ncbi.nlm.nih.gov/geo/query/acc.cgi?acc=GSE126243
ZFP57 - https://www.ncbi.nlm.nih.gov/geo/query/acc.cgi?acc=GSE123942
ZMYM2 - https://www.ncbi.nlm.nih.gov/geo/query/acc.cgi?acc=GSE119820
ATF7IP - https://www.ncbi.nlm.nih.gov/bioproject/PRJNA664286

Newly-generated genomics data has been deposited to NCBI GEO under the following accession number GSE176461;
Newly-generated mass spectrometry proteomics data have been deposited to the ProteomeXchange Consortium via the dataset identifier PXD034918.

Field-specific reporting

Please select the one below that is the best fit for your research. If you are not sure, read the appropriate sections before making your selection.

☒ Life sciences ☐ Behavioural & social sciences ☐ Ecological, evolutionary & environmental sciences

For a reference copy of the document with all sections, see nature.com/documents/nr-reporting-summary-flat.pdf

Life sciences study design

All studies must disclose on these points even when the disclosure is negative.

Sample size	No statistical method was used to pre-determine sample size.
Data exclusions	Genomic regions with low-mappability scores (defined by ENCODE) were excluded from further analysis.
Replication	All experiments were confirmed by replication at least once with independent cell lines, except for the EpiTF CRISPR screen which itself was intended to be used as an independent replication for the screens performed with the ChromMM library. The exact handling of replicates is depicted in figure panels or the methods section.
Randomization	Not relevant for this genomics experiments performed here. Samples were allocated to either wild type or mutant and processed in parallel. Form MS measurements, block-randomization was applied.
Blinding	Blinding not relevant for this study. All samples were processed through identical analysis pipelines in parallel.

Reporting for specific materials, systems and methods

We require information from authors about some types of materials, experimental systems and methods used in many studies. Here, indicate whether each material, system or method listed is relevant to your study. If you are not sure if a list item applies to your research, read the appropriate section before selecting a response.

Materials & experimental systems

n/a	Involved in the study	
☐	☒	Antibodies
☐	☒	Eukaryotic cell lines
☒	☐	Palaeontology and archaeology
☒	☐	Animals and other organisms
☒	☐	Human research participants
☒	☐	Clinical data
☒	☐	Dual use research of concern

Methods

n/a	Involved in the study	
☐	☒	ChIP-seq
☐	☒	Flow cytometry
☒	☐	MRI-based neuroimaging

Antibodies

Antibodies used	The following antibodies were used for FACS:<br>monoclonal APC-conjugated anti-CD90.1 Invitrogen #17-0900-82 - clone HIS51<br><br>The Following antibodies were used for ChIP:<br>polyclonal anti-H3K9me3 abcam #ab8898<br>polyclonal anti-H3K4me2 Diagenode, C15410035<br>polyclonal anti-ATF7IP Bethyl, A300-169A<br>polyclonal anti-ZMYM2 Bethyl, A301-711A<br><br>The Following antibodies were used for WB:<br>anti-LaminB1 Santa Criuz, sc-3019<br>anti-ATF7IP Bethyl, A300-169A<br>anti-ZMYM2 Bethyl, A301-711A<br>Pierce goat anti-rabbit IgG (H+L) HRP-conjugated (ThermoFischer, 31466),<br>Pierce goat anti-mouse IgG (H+L) HRP-conjugated (ThermoFischer, 31431).
Validation	All antibodies are commercial antibodies and validated by the provider and numerous publications.<br><br>FACS<br>monoclonal APC-conjugated anti-CD90.1 Invitrogen #17-0900-82 - clone HIS51:<br>https://www.thermofisher.com/antibody/product/CD90-1-Thy-1-1-Antibody-clone-HIS51-Monoclonal/17-0900-82<br><br>ChIP<br>polyclonal rabbit anti-H3K9me3 (Abcam, ab8898, 5µg/IP): http://www.histoneantibodies.com/FinalArrayData/H3K9me3/<br>polyclonal anti-H3K4me2 Diagenode, C15410035 (5 µg/IP): http://www.histoneantibodies.com/FinalArrayData/H3K4me2/<br>anti-ATF7IP Bethyl, A300-169A:<br>https://www.thermofisher.com/antibody/product/MCAF-Antibody-Polyclonal/A300-169A-M<br>anti-ZMYM2 Bethyl, A301-711A:<br>https://www.fortislife.com/products/primary-antibodies/rabbit-anti-znf198-antibody/BETHYL-A301-711<br><br>WB<br>anti-LaminB1 Santa Criuz, sc-3019:<br>https://www.scbt.com/p/lamin-b1-antibody-c-12<br>anti-ATF7IP Bethyl, A300-169A:<br>https://www.thermofisher.com/antibody/product/MCAF-Antibody-Polyclonal/A300-169A-M<br>anti-ZMYM2 Bethyl, A301-711A:<br>https://www.fortislife.com/products/primary-antibodies/rabbit-anti-znf198-antibody/BETHYL-A301-711

Eukaryotic cell lines

Policy information about cell lines

Cell line source(s)	TC-1: Lienert et al., 2011. DOI: 10.1038/nature10716<br>HEK293T: ATCC RRID: CVCL_0063<br>MEL: Feng et al., 2001 DOI:10.1128/MCB.21.1.298-309.2001
Authentication	Genotype confirmed by PCR/Sanger sequencing and Western blot.
Mycoplasma contamination	Tested negative
Commonly misidentified lines<br>(See ICLAC register)	No commonly misidentified cell lines were used.

ChIP-seq

Data deposition

☒ Confirm that both raw and final processed data have been deposited in a public database such as GEO.

☐ Confirm that you have deposited or provided access to graph files (e.g. BED files) for the called peaks.

Data access links<br>*May remain private before publication.*	https://www.ncbi.nlm.nih.gov/geo/query/acc.cgi?acc=GSE176461
Files in database submission	ChIP-seq data for H3K9me3 in ES cells
Genome browser session<br>(e.g. UCSC)	no longer applicable

Methodology

Replicates	one replicate per clone performed
Sequencing depth	Over 20 Mio reads aligned once after deduplication and filtering of reads with MAPQ >40
Antibodies	polyclonal anti-H3K9me3 Bethyl, A300-169A (5 µg/IP)
Peak calling parameters	No peaks were called on the generated H3K9me3 data
Data quality	Sequencing quality was assessed using QuasR qQCReport()
Software	trim_galore 0.6.6<br>QuasR 1.30.0

Flow Cytometry

Plots

Confirm that:

☒ The axis labels state the marker and fluorochrome used (e.g. CD4-FITC).

☒ The axis scales are clearly visible. Include numbers along axes only for bottom left plot of group (a 'group' is an analysis of identical markers).

☒ All plots are contour plots with outliers or pseudocolor plots.

☒ A numerical value for number of cells or percentage (with statistics) is provided.

Methodology

Sample preparation	Cells were harvested by trypsinisation and resuspended in 2 % FCS in DPBS for analysis. If necessary, cells were incubated with monoclonal APC-conjugated anti-CD90.1 Invitrogen #17-0900-82 (1 µl per 15 million cells) for 30 min at 4 ˚C. Samples were analysed for eGFP, mScarlet, and APC expression by flow cytometry on a FACSCanto II (BD Biosciences), LSR Fortessa (BD Biosciences), or FACSAria III cell sorter (BD Biosciences).
Instrument	FACSCanto II (BD Biosciences), LSR Fortessa (BD Biosciences), or FACSAria III cell sorter (BD Biosciences).
Software	Data was aquired with the BD FACSDiva Software (BD Biosciences) and analysed with FlowJo 10.7
Cell population abundance	Purity was assessed for the first sample of a sort.
Gating strategy	Gating was performed based on WT cells gated for single cells, using forward scatter area (FSC-A) vs. side scatter area (SSC-A), followed by FSC-A vs. forward scatter height (FSC-H). Gating for eGFP, mScarlet, and APC was done with the help of single-stained or single-fluorescent cells.

☒ Tick this box to confirm that a figure exemplifying the gating strategy is provided in the Supplementary Information.
