# Supplementary material for: DNA sequence and chromatin modifiers cooperate to confer epigenetic bistability at imprinting control regions
Source: Nat Genet. 2022 Nov 4;54(11):1702–10. doi: 10.1038/s41588-022-01210-z (PMC9649441; doi:10.1038/s41588-022-01210-z)
Supplement: Supplementary file 3 [file 41588_2022_1210_MOESM4_ESM.pdf]

Source File for Extended Data Figure 1b

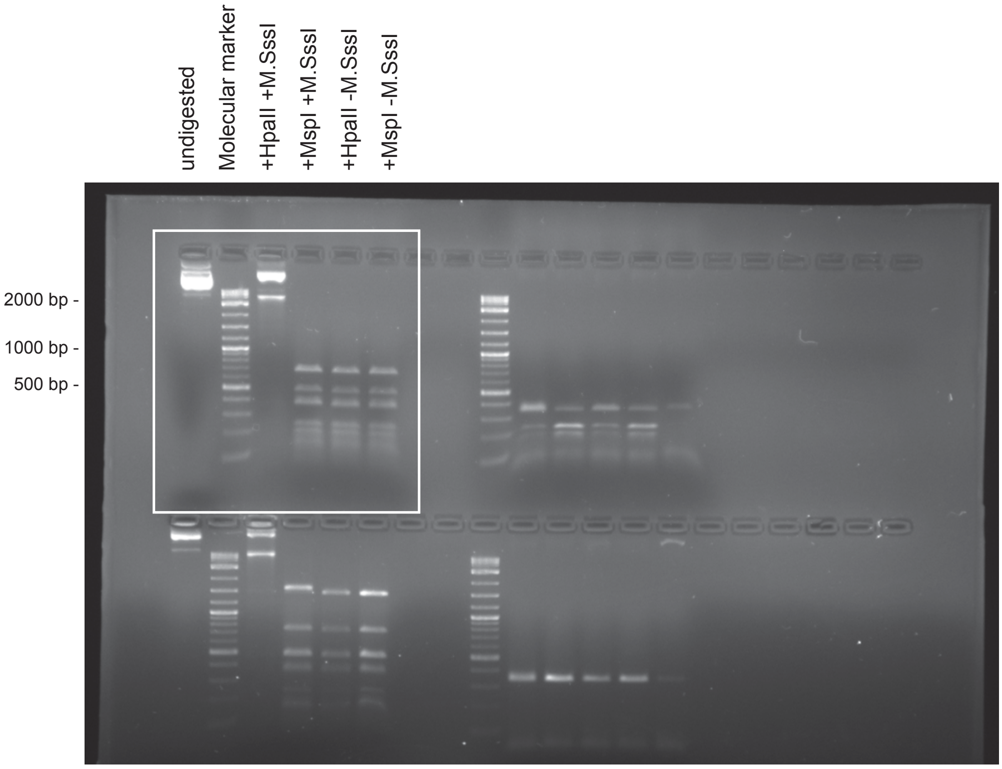

HpaII / MspI restriction digest agarose gel
